# The mental health impact of the 2023 earthquakes on the Syrian population: cross-sectional study

**DOI:** 10.1192/bjo.2023.598

**Published:** 2023-12-01

**Authors:** Jameel Soqia, Amjad Ghareeb, Rana Hadakie, Kinda Alsamara, David Forbes, Kenda Jawich, Alaa Al-Homsi, Ameer Kakaje

**Affiliations:** Faculty of Medicine, Damascus University, Syria; Department of Biochemistry and Microbiology, Faculty of Pharmacy, Damascus University, Syria; Centre for Arab and Islamic Studies, Australian National University, Canberra, Australia; Phoenix Australia – Centre for Posttraumatic Mental Health, Department of Psychiatry, University of Melbourne, Australia; Faculty of Medicine, Damascus University, Syria; and University Hospital Geelong, Barwon Health, Geelong, Australia

**Keywords:** Anxiety, depression, natural disaster, earthquake, mental health

## Abstract

**Background:**

Natural disasters have a significant impact on the mental health of affected populations. The February 2023 earthquakes in Syria and Turkey caused widespread devastation.

**Aims:**

To explore the mental health impact of the earthquakes in Syria on the population across areas differentially damaged by the disaster.

**Method:**

This cross-sectional study conducted in Syria included 1406 adults recruited via social media platforms 1 month after the February 2023 earthquakes. Demographic information, earthquake exposure questions, the PTSD Checklist for DSM-5 (PCL-5: for probable post-traumatic stress disorder, PTSD), the Patient Health Questionnaire-9 (PHQ-9: for probable depression) and the seven-item Generalized Anxiety Disorder scale (GAD-7: for probable anxiety) were included to compare outcomes across areas severely, moderately and slightly damaged by the earthquakes.

**Results:**

Probable PTSD and GAD rates were higher in the severely (57.9 and 57.3% respectively) and moderately damaged regions (55.4 and 56.3% respectively) than in the slightly damaged regions (44.6 and 48.3% respectively) (PTSD: *P* < 0.001, GAD: *P* = 0.005). More participants in severely damaged regions (60.6%) reported symptoms of depression compared with moderately (53.1%) and slightly damaged (50.8%) regions (*P* = 0.003). Poorer mental health outcomes were associated with being female, single, younger, having a damaged or destroyed house, seeing something tragic in person and hearing tragic stories. Seeing something tragic on social media was not statistically significant.

**Conclusions:**

This study highlights the higher prevalence of probable mental disorders in areas with more severe earthquake damage, with over 50% of the population reporting probable PTSD, depression or anxiety. The study also suggests a significant cumulative effect of these earthquakes on an already trauma- and disaster-affected population.

The aftermath of a natural disaster can leave deep and lasting scars on the mental health of those affected. From the rubble of collapsed buildings to the loss of loved ones, survivors of such events can experience a range of mental health problems, including post-traumatic stress disorder (PTSD), depression and anxiety.^1–4^ The Turkey–Syria earthquakes of February 2023 were no exception, bringing widespread devastation to a region already reeling from years of conflict, as Syria has been suffering from war and unrest for over 10 years, with millions displaced, internally and externally, and many losing their homes and families.^5–7^

On 6 February 2023, Syria and Turkey were struck by two powerful earthquakes, with magnitudes of 7.8 and 7.5, followed on 20 February 2023 by an earthquake of magnitude of 6.4, directly affecting over 26 million people and causing destruction to homes, infrastructure and facilities.^5,8–12^ The death toll has exceeded 40 000, with many more displaced, seeking shelter in temporary accommodation and schools. In Syria specifically, according to UNHCR, the UN Refugee Agency, it is estimated that more than 8.8 million people were affected by the earthquakes, with over 385 000 needing emergency response, and over 1400 deaths and 2350 injuries reported by the Syrian Ministry of Health.^11,13^ Trauma-related mental disorders, and PTSD in particular, can have long-lasting effects and result in significant disability, with profound effects on quality of life.^14^ Recognising and addressing these trauma-related disorders is crucial to mitigating their potential long-term effects on emotional well-being and functional capacity. Although media coverage focused on the immediate aftermath of the disaster, there has yet to be any study of the mental health impacts. This study examines the probability of having PTSD, depression and anxiety among survivors in Syria of the Turkey–Syria earthquakes, 1 month after the event, across areas differentially damaged by the disaster. It aims to shed light on a critical but often overlooked aspect of disaster recovery, identifying the impact of the disaster on an already trauma-affected population and the need for ongoing support beyond the immediate emergency status.^13^

## Method

### Participants and sampling

This was a nationwide cross-sectional study in Syria conducted 1 month after the devastating Syrian–Turkish earthquakes. The study sample was 1406 individuals who responded between 15 March and 1 April 2023 to an invitation via social media platforms (Facebook and Telegram) distributed within governorates to participate in the study and complete a validated structured self-report questionnaire. Individuals aged 18 years or older were eligible to participate. This study followed Strengthening the Reporting of Observational Studies in Epidemiology (STROBE) guidelines for cross-sectional studies.

Syria is divided into 14 governorates, which are administrative areas that contain the cities and other regions. The governorates in Syria most badly damaged by the earthquakes were Aleppo, Idleb, Latakia, Tartous and Hama.^15^ In this study, we divided the governorates into three categories by level of earthquake-related damage, based on a World Bank report of the impact of the disaster:^15^ severely damaged, moderately damaged and slightly damaged. This categorisation was based on the percentage of total damage in the governorates as recorded in the report. Total damage included direct physical damage to residential and non-residential buildings and contents and to infrastructure. Our study covers two severely damaged regions (Aleppo and Latakia), two moderately damaged regions (Hama and Tartous) and nine slightly damaged regions (Homs, Raqqa, Deir ez-Zur, Al Hasakah, Rif Dimashq, Damascus, As-Suwayda, Daraa and Al Qunaitra). Idleb, a severely damaged region, was excluded from the study, given limitations in accessing participants due to very limited electricity and internet access.

### Measures

The survey questionnaire was divided into five sections.

#### Demographics

Participants were asked to give their age, gender, educational level, marital status, work status and the governorate in which they were living when the earthquakes occurred.

#### Earthquake exposure questions

To measure exposure to the earthquake, the study used questions from a modified version of a four-item instrument developed by Shi et al, which evaluated the casualties of family members, house damage, property loss and witnessing tragic scenes.^16^ We added a fifth item to account for exposure via media or social media, which was used in previous research.^17^ We added a further two questions regarding loss of a family member in the earthquake and one or more family member(s) being trapped under rubble. Thus, this section of the questionnaire included seven items, with each individual question evaluated independently.

#### Probable post-traumatic stress disorder

The PTSD Checklist for DSM-5 (PCL-5) was used. This standardised self-report measure, which assesses the symptoms of PTSD based on the diagnostic criteria outlined in DSM-5, has good psychometric properties.^18–20^ The PCL-5 consists of 20 items that assess the severity of PTSD symptoms in the past month. The Arabic version was validated in a previous study and showed robust psychometric properties for evaluating PTSD.^18^ A cut-off between 31 and 33 is usually recommended. A conservative cut-off of 33 was used in the present study.^18^ The present study obtained a Cronbach's alpha of 0.93 for the PCL-5, suggesting strong internal reliability.

#### Probable depression

The nine-item Patient Health Questionnaire (PHQ-9) was used to screen for the presence of depression. This is a valid tool across multiple settings and widely used.^21,22^ Respondents were asked to report how often they had experienced various depression-related symptoms in the past 2 weeks, with responses on a four-point Likert scale ranging from ‘not at all’ (0) to ‘nearly every day’ (3).^4^ Criteria for a probable major depressive episode were met if the score was ≥10, which is consistent with previous recommendations.^21,22^ The Arabic version was validated in a previous study and showed good psychometric properties for evaluating depressive symptoms.^23^ The present study obtained a Cronbach's alpha of 0.89 for the PHQ-9, suggesting strong internal reliability.

#### Probable generalised anxiety disorder

To evaluate the presence of anxiety, the seven-item Generalized Anxiety Disorder scale (GAD-7) was employed, which has been validated in the general population for its psychometric properties.^24–26^ This tool has been used in previous research to measure generalised anxiety disorder in individuals exposed to disasters. Each question on the GAD-7 is scored from 0 (not at all) to 3 (nearly every day), with a maximum possible score of 21. To improve the sensitivity and specificity of anxiety detection, our study utilised a cut-off of ≥10 as an indication of an anxiety problem, consistent with previous recommendations.^24–26^ The Arabic version was validated in a previous study and showed good psychometric properties.^23^ The present study obtained a Cronbach's alpha of 0.93 for the GAD-7, suggesting strong internal reliability. The time needed to complete the questionnaire was approximately 10–12 min.

### Ethics approval and consent to participate

The authors assert that all procedures contributing to this work comply with the ethical standards of the relevant national and institutional committees on human experimentation and with the Helsinki Declaration of 1975, as revised in 2008. All procedures involving human subjects/patients were approved by the local ethics committee of Damascus University, Faculty of Medicine (approval number 28 442 ني ). Written informed consent was obtained from all participants. This study did not include participants younger than 18 years old.

### Statistical analysis

Google Forms was used to collect data online. Prevalence rates were calculated based on the recommended cut-offs for probable PTSD, depression and GAD. Differences in prevalence rates of probable PTSD, depression and GAD across the three differentially damaged regions were assessed using chi-squared analyses. Separate logistic regressions were conducted to identify predictors of probable PTSD, depression and GAD respectively. In each regression gender, age, work and marital status and the highest level of education were entered as predictors at step 1 and factors reflecting earthquake exposure were entered at step 2.

## Results

### Demographic characteristics

In total, 1406 participants completed the questionnaire. Overall, probable prevalence of PTSD was 51.2% (*n* = 720), probable prevalence of depression was 54.8% (*n* = 770) and probable prevalence of generalised anxiety disorder was 52.9% (*n* = 744). As outlined in Table 1, the mean age of participants was 28.62 years (s.d. = 8.86), 77.7% were female (*n* = 1093), 90.7% (*n* = 1275) had completed tertiary education, 66.8% (*n* = 939) were single and 29.2% (*n* = 410) reported being in full-time work or study. Further details, characteristics and differences in participants across the three regions are outlined in Table 1.

**Table 1:** Demographic characteristics and earthquake trauma exposure in regions severely, moderately and slightly damaged by the earthquakes

	Region		
	Severely damaged	Moderately damaged	Slightly damaged
Gender (female), *n* (%)	385 (74%)	179 (79.9%)	529 (79.9%)
Age, years: mean (s.d.)	27.95 (8.42)	29.12 (8.51)	28.99 (9.28)
Tertiary education, *n* (%)	480 (92.3%)	206 (92%)	589 (89%)
Full-time work/study, *n* (%)	150 (28.8%)	80 (35.7%)	180 (27.2%)
Single, *n* (%)	435 (65.7%)	148 (66.1%)	356 (68.5%)
Lost family member to earthquake, *n* (%)	21 (4%)	2 (0.9%)	8 (1.2%)
Family member(s) trapped under the rubble of earthquake, *n* (%)	14 (2.7%)	0	10 (1.5)
Damage to the house you live in by earthquake, *n* (%)	85 (16.3%)	23 (10.3%)	15 (2.3%)
Damage to other property by earthquake, *n* (%)	31 (6%)	3 (1.3%)	14 (2.1%)
Witnessed something tragic from earthquake, *n* (%)	193 (37.1%)	27 (12.1%)	41 (6.2%)
Watched something tragic about earthquake on media, *n* (%)	456 (87.7%)	192 (85.7%)	591 (89.3%)
Heard tragic stories about earthquake from someone, *n* (%)	451 (86.7%)	178 (79.5%)	534 (80.7%)

In the severely damaged regions 16.3% (*n* = 85) of participants reported that their houses had been damaged by the earthquake, in contrast to 10.3 and 2.3% in moderately and slightly damaged regions respectively. Furthermore, 37.1% (*n* = 193) of participants from severely damaged regions personally witnessed tragic scenes from the earthquake, as opposed to 12.1 and 6.2% from moderately and slightly damaged regions respectively.

### Prevalence of probable mental health outcomes

Table 2 presents the frequencies of reported probable PTSD, depression and GAD by regional level of earthquake damage. More participants from the severely (57.9%) and moderately damaged (55.4%) regions reported probable PTSD than those from the slightly damaged (44.6%) regions (χ^2^(2, *n* = 1406) = 22.52; *P* < 0.001). For depression, more participants from the severely damaged regions (60.6%) reported probable depression, compared with 53.1 and 50.8% from the moderately and slightly damaged regions respectively (χ^2^(2, *n* = 1406) = 11.63; *P* = 0.003).

**Table 2:** Prevalence of probable post-traumatic stress disorder (PTSD), depressive disorder and generalised anxiety disorder in regions severely, moderately and slightly damaged by the earthquakes^a^

	Severely damaged, *n* (%)	Moderately damaged, *n* (%)	Slightly damaged, *n* (%)	*P*	χ^2^
PTSD	301 (57.9%)	124 (55.4%)	295 (44.6%)	<0.001**	22.524
Depression	315 (60.6%)	119 (53.1%)	336 (50.8%)	0.003*	11.630
Generalised anxiety disorder	298 (57.3%)	126 (56.3%)	320 (48.3%)	0.005*	10.592

a. PTSD was measured by the PTSD Checklist for the DSM-5 (PCL-5), depression by the nine-item Patient Health Questionnaire (PHQ-9) and generalised anxiety disorder by the seven-item General Anxiety Disorder scale.

*P < 0.05, **P < 0.001.

Similarly, participants from the severely (57.3%) and moderately damaged (56.3%) regions were more likely to report probable GAD than participants from the slightly damaged regions (48.3%) (χ^2^(2, *n* = 1406) = 10.59; *P* = 0.005).

### Gender and mental health outcomes

Table 3 presents the rates of mental health outcomes by gender. Females were significantly more likely than males to develop probable PTSD (53.9% *v*. 41.9%; OR = 1.62, 95% CI 1.25–2.09; *P* < 0.001) and probable depression (56.7% *v.* 47.9%; OR = 1.42, 95% CI 1.10–1.83; *P* = 0.006). However, there were no significant differences between males and females for probable GAD.

**Table 3:** Unadjusted odds ratios between males and females for probable post-traumatic stress disorder (PTSD), depressive disorder and generalised anxiety disorder^a^

	Males (reference), *n* (%)	Females, *n* (%)	OR (95% CI)
PTSD	132 (41.9%)	589 (53.9%)	1.62 (1.25–2.09)**
Depression	150 (47.9%)	620 (56.7%)	1.42 (1.10–1.83)*
General anxiety disorder	152 (48.6%)	592 (54.2%)	1.25 (0.97–1.61)

a. PTSD was measured by the PTSD Checklist for the DSM-5 (PCL-5), depression by the nine-item Patient Health Questionnaire (PHQ-9) and generalised anxiety disorder by the seven-item General Anxiety Disorder scale.

*P < 0.05, **P < 0.001.

### Predictors of mental health outcomes

Table 4 presents the adjusted odds ratios for probable PTSD, depression and GAD using binary logistic regression. Probable PTSD was predicted by female gender, younger age, being single, earthquake damage to their houses, personally witnessing tragic scenes from the earthquake and hearing tragic stories caused by earthquake. Probable depression was predicted by female gender, earthquake damage to their houses, personally witnessing tragic scenes from the earthquake and hearing tragic stories caused by earthquake. Probable GAD was predicted by earthquake damage to their houses, personally witnessing tragic scenes from the earthquake and hearing tragic stories caused by earthquake but by none of the demographic variables. Numbers of participants who lost loved ones in the earthquake were too low to include in the analyses (*n* < 10).

**Table 4:** Binary regressions for adjusted odds ratios of probable post-traumatic stress disorder (PTSD), depressive disorder and generalised anxiety disorder for multiple variables^a^

	Adjusted OR (95% CI)		
	PTSD	Depression	Generalised anxiety disorder
Gender (female)	1.76 (1.33–2.32)**	1.47 (1.12–1.92)**	1.28 (0.98–1.67)
Age	0.98 (0.96–0.99)*	1.00 (0.99–1.02)	1.01 (0.99–1.02)
Tertiary education	0.77 (0.51–1.16)	0.84 (0.56–1.24)	0.74 (0.50–1.11)
Full-time work/study	0.92 (0.70–1.21)	0.84 (0.64–1.10)	0.98 (0.75–1.28)
Single	0.66 (0.49–0.86)*	1.04 (0.78–1.40)	0.78 (0.58–1.04)
Lost family to earthquake	1.67 (0.73–3.81)	1.33 (0.59–2.99)	1.59 (0.71–3.56)
Family trapped under the rubble of earthquake	1.05 (0.42–2.60)	1.42 (0.57–3.53)	1.61 (0.65–3.99)
Damage to the house you live in by earthquake	4.07 (2.49–6.65)**	2.57(1.63–4.05)**	2.92 (1.85–4.61)**
Damage to properties by earthquake	0.65 (0.34–1.25)	0.99 (0.51–1.90)	0.74 (0.39–1.41)
Witnessed something tragic from earthquake	1.74 (1.28–2.36)**	1.60 (1.18–2.17)*	1.55 (1.14–2.09)*
Watched something tragic about earthquake on media	1.27 (0.86–1.86)	0.77 (0.53–1.12)	1.10 (0.76–1.59)
Heard tragic stories about earthquake from someone	2.35 (1.68–3.27)**	2.19 (1.59–3.03)**	1.83 (1.33–2.53)*

a. PTSD was measured by the PTSD Checklist for the DSM-5 (PCL-5), depression by the nine-item Patient Health Questionnaire (PHQ-9) and generalised anxiety disorder by the seven-item General Anxiety Disorder scale.

*P < 0.05, **P < 0.001.

## Discussion

The present study is the first to investigate the prevalence of probable PTSD, depression and GAD among survivors of the February 2013 earthquakes in Syria, 1 month after the event, and through the lens of differential regional damage caused by the earthquakes (severe, moderate and slight damage).

This study found significantly higher rates of both probable PTSD and probable GAD in the severely (57.9 and 57.3% respectively) and moderately (55.4 and 56.3% respectively) damaged regions compared with the slightly (44.6 and 48.3% respectively) damaged regions. In addition, the study identified higher rates of probable depression in the severely (60.6%) compared with the moderately (53.1%) and slightly (50.8%) damaged areas. Importantly, the rates of these probable disorders in the slightly damaged areas are reasonably consistent with (although a little higher than) the rates identified in prevalence studies in Syria over the 4 years prior to this recent disaster (35.6% in 2019 and 42.6% in 2021), which were estimated using post-traumatic stress symptoms with a diagnosis of probable PTSD when scoring two symptoms or more.^6,7^ These previous studies used a similar methodology and sampling to the present study and are hence of relevance for comparison. This consistency provides a reference point for the impact of this subsequent severe disaster on an already highly trauma-affected population. That rates in the slightly damaged regions in the current study are slightly higher than these previous national rates might be understood in the context of the fear that was pervasive across Syria during and after the earthquakes relating to possible future risk. Hence there is still an added exposure of the recent earthquakes despite being in regions with comparatively little earthquake damage.

Of significant concern is that these pre-earthquake prevalence rates for mental health problems were already exceedingly high and warranted urgent and substantive attention. These previous studies, along with the present study, suggest how the cumulative effect of subsequent traumas can still increase mental health suffering and distress. Through this study we have not only observed the differential mental health impact of the recent earthquakes on the Syrian population based on level of regional damage, but also identified the compounding effect of the disaster on a population with significant pre-existing trauma load and mental healthcare need. For example, 15.3 million people prior to the earthquakes in Syria were in need of humanitarian assistance, with 4.1 million living in catastrophic conditions.^11^ Despite all efforts, affected communities remained highly vulnerable and this study highlights the ongoing support needed to develop a response beyond the immediate emergency phase.^13^

The findings of this study are consistent with previous earthquake research that reported rates of 61% for PTSD among South East Anatolian females after the 2004 earthquake^27^ and 58.3% among adolescents affected by the 2016 Aceh earthquake.^28^ The findings of this study are also consistent with other earthquake studies in the area (survivors of the Marmara earthquake in Turkey in August 1999) demonstrating substantially higher rates of PTSD (23%) and comorbidity with depression (16%) at the epicentre, compared with safer cities (14 and 8% respectively in Istanbul).^29^ Data from the Marmara earthquake also showed elevation in rates of psychological distress in the safe cities, consistent with our findings of some elevation in distress even in the slightly damaged regions.^30^ These rates of PTSD are higher than those reported in a systematic review of disaster survivors overall, where rates generally ranged between 30 and 40%, but varied considerably based on the nature of exposure, context and measurement methodologies.^31^ Of great concern in the findings in our study is not only the current mental health impact of these earthquakes and their compounding of the mental health burden from prior trauma, but also recognition from previous research that the prevalence of these mental health problems often remains elevated 3 or more years after the disaster.^32^

In the examination of the predictors of mental health outcomes, this study identified that, consistent with existing literature, females were at higher risk for the development of probable PTSD and depression,^6,7,28,33,34^ although this finding was not evident for probable GAD. In terms of specific disaster-related predictors, experiencing damage to one's house, personally witnessing tragic scenes and directly hearing tragic stories about the earthquake all independently increased the prediction of probable PTSD, depression and GAD. Loss of other properties (not one's house) and exposure via media did not increase the risk for any of these disorders. Hence it may be understood that more direct personal loss or direct exposure to suffering of others were predictive. Importantly, the number of individuals in our sample who lost loved ones in the earthquakes was too low to include in the analyses; however, based on existing research, we would expect higher rates among all those who had personally suffered these tragic losses.

### Implications

In considering the implications of the findings of this study it needs to be recognised that the added burden of mental illness following this event occurs in a country where the availability of physical and mental healthcare was already extremely low. Furthermore, in terms of specialist mental health services, over the past decade there have been fewer than 100 psychiatrists across the country. In 2018 there was less than 1 psychiatrist per 100 000 population and more than 90% of patients were unattended and untreated, a situation further complicated by severe lack of resources and funding and continued public stigma and self-stigma relating to recognition of mental illness and service seeking.^6,35^

This paper has identified that although mental suffering was most prevalent in regions most damaged by the earthquakes, the very high rates of probable PTSD, depression and anxiety across all areas, including the slightly damaged regions, indicates the importance of not focusing support initiatives on the survivors of recent events in the absence of attention to the wider population across the country suffering the mental health effects of multiple traumas and disasters and deprivation in the meeting of basic needs. This paper identifies the mental health burden and the extent of risk following these recent earthquakes in a directly affected population who could benefit from targeted mental health interventions such as psychological first aid, cognitive–behavioural therapy and other evidence-based interventions.^36^ However, most critically, systemic initiatives are required that address both the mental health and basic care needs of people across all regions. A team of psychiatrists, general physicians and psychotherapists volunteered free of charge to support those who were most affected by the earthquakes in Syria^37^ but a more sustainable plan is needed for the long term.

### Limitations

The study has several limitations that should be considered when interpreting the results. Most importantly, the need to conduct the study online rather than in-person was a major limiting factor, as people who had lost their home and were likely most affected by the earthquakes would be the most difficult to access for a number of reasons, including logistics of access to the internet and social media. This was most apparent for Idleb, which was excluded from the study for these reasons. In addition, endorsements of earthquake-related questions such as losing loved ones in the earthquake were very low in what must be an underrepresentation of this group, given the known death toll. ^5,8–11,13^ All of these factors would potentially contribute to the probable mental health burden reported in this study (albeit already high), giving an underestimate of the actual mental health burden. Although previous studies cited in this paper^6,7,11,13^ outlined the mental health burden of trauma prior to the earthquakes, and participants were instructed to respond to the survey questions with the recent earthquakes in mind, this study does not include formal control for prior trauma.

This study was cross-sectional in nature, which limits the ability to establish causal relationships between the predictors and mental health outcomes. Moreover, the study relied on self-reported measures of mental health outcomes, which may be subject to biases, given their subjective rather than objective methodology. In addition, this study did not use a randomised stratified methodology. Furthermore, the study did not evaluate other potential factors, such as social support, coping strategies and access to mental health services, which may influence mental health outcomes. Finally, this study reported only on the mental health burden on adults, and it did not include children and adolescents.

## Data Availability

The data that support the findings of this study are available from the corresponding author, on reasonable request.
